# Mortality involving non-rheumatic valvular heart disease and atrial fibrillation in the United States: Long-term trends and implications

**DOI:** 10.1097/MD.0000000000051020

**Published:** 2026-10-09

**Authors:** Ibtissam ElGhazzani, Youssef Ramadan, Alyaa Ahmed Ibrahim, Maryam Saghir, Muhammad Sarim Azad Khan, Karim Othman Abdelnaim, Moustafa Ahmed Elsayed, Abdul Samad, Amr Arafa, Krish Patel, Eshal Saghir, F.N.U. Sahil, Maha Sajjad, Yahya Samadi, Mohamed Fawzi Hemida

**Affiliations:** a Faculty of Medicine of Tunis, Tunis, Tunisia; b Cardiovascular Research Group, Fawzi Research Network, Alexandria, Egypt; c Faculty of Medicine, October 6 University, Giza, Egypt; d Faculty of Medicine, Alexandria University, Alexandria, Egypt; e Department of Medicine, Jinnah Sindh Medical University, Karachi, Pakistan; f Department of Medicine, Combined Military Hospital, Lahore Medical College, Lahore, Pakistan; g Faculty of Medicine, Ain Shams University, Cairo, Egypt; h Faculty of Medicine, Tanta University, Tanta, Egypt; i Department of Medicine, Dow University of Health Sciences, Karachi, Pakistan; j Department of Medicine, C. U. Shah Medical College, Surendranagar, Gujarat, India; k Department of Medicine, Liaquat University of Medical & Health Sciences, Jamshoro, Pakistan; l Department of Medicine, King Edward Medical University, Lahore, Pakistan; m Department of Medicine, Kateb University, Kabul, Afghanistan; n Department of Internal Medicine, University of Vermont, Burlington, Vermont; o Department of Cardiovascular Medicine, American Society for Inclusion, Diversity, and Equity in Healthcare (ASIDE), Lewes, Delaware.

**Keywords:** atrial fibrillation, cardiovascular mortality, death certificate data, mortality trends, non-rheumatic valvular heart disease, population-based study, United States, valvular heart disease

## Abstract

Non-Rheumatic valvular heart disease (NRVHD) and atrial fibrillation (AF) are major contributors to cardiovascular mortality in the U.S. Their coexistence increases the risk of stroke, heart failure, and death.This study examines national trends in deaths involving both NRVHD and AF in the United States from 1999 to 2023. Centers for disease control and prevention - Wide-Ranging Online Data for Epidemiologic Research data (1999–2023) were analyzed for adults with AF (I48) and NRVHD (I34–I36) as contributing causes of death. Age-adjusted mortality rates (AAMRs) were calculated per 100,000 population. Joinpoint regression analysis was used to determine average and annual percent change (AAPC) and annual percent change. From 1999 to 2023, a total of 141,846 deaths in the U.S. involved both AF and NRVHD. The AAMR increased from 2.98 in 1999 to 7.30 in 2023 (AAPC: 4.17; *P* < .001). Males had higher overall AAMR (5.31) (AAPC: 4.73) than females (4.02) (AAPC: 3.58). Non-hispanic Whites had the highest overall AAMR (5.17) while Hispanics had an AAMR of (1.88). Regionally, West had the highest overall AAMR (5.73), followed by the Northeast (4.81), the Midwest (4.79), and then the South (3.48). Majority of the deaths occurred in inpatient medical facilities (49,170 deaths, 35.0%). The age group 65 + showed the highest mortality (crude mortality rate: 11.93). Rural areas had higher overall AAMR (4.36) compared to urban areas (4.10). Deaths involving both AF and NRVHD increased from 1999 to 2023, with greater burden among older adults, males, rural areas, the West region, and non-Hispanic White population.

## 1. Introduction

Patterns within the cardiovascular disease (CVD) spectrum have changed over the past 2 decades.^[1]^ Mortality from specific cardiac diseases, such as valvular heart disease and AF, has become apparent.^[2]^ Non-rheumatic valvular heart disease (NRVHD) and AF frequently coexist and have important pathophysiological interactions. Valvular dysfunction can promote atrial remodeling and predispose to AF, while AF may further impair cardiac hemodynamics and increase the risk of heart failure and thromboembolic complications.^[3]^ Therefore, examining deaths involving both conditions provides insight into their combined mortality burden and clinical impact. They frequently coexist as causes of cardiovascular death and morbidity. Hence, the number of deaths worldwide from CVD has increased from 12.4 million in 1990 to 19.8 million in 2022.^[2]^

This increase in mortality rate is due to the world’s population aging and the contributions of several risks, involving metabolic, behavioral, and environmental factors.^[4]^ NRVHD comprises degenerative lesions such as aortic stenosis or calcification, and mitral regurgitation, excluding rheumatic heart pathology.^[5]^ Moreover, there is a growing prevalence of valvular disease, which affects more than 2.5% of the United States (US) population.^[6]^ In particular, among patients with CVD, there were 29.2 million cases of NRVHD. Of these patients with NRVHD, 13.6 million had aortic valve calcification, 15.6 million had degenerative mitral valve disease, and 0.01 million had other valve diseases.^[2]^

The incidence rate of NRVHDs increases after the age of 45, peaks between the ages of 65 and 69, and then gradually decreases. The affected age group makes it comorbid with AF, which is also highly prevalent and affects more than 60 million older adult individuals.^[7,8]^

Notably, the actual AF prevalence is higher, since many people with AF go undiagnosed until they exhibit symptoms or anembolic ischemic stroke.^[9]^ AF is characterized by unorganized atrial electrical activity leading to ineffective or incomplete atrial contraction and irregular response of the ventricles.^[10]^

The coexistence of AF and NRVHD carries significant strain. Valvular dysfunction promotes atrial enlargement and fibrosis. As a result, AF develops, leading to hemodynamic overload on the diseased valve and increasing the risk of thromboembolic complications. Their coexistence worsens outcomes and complicates their management.^[11]^ Therapeutic decisions often involve long-term anticoagulation and arrhythmia control with the optimal timing of surgical or transcatheter valve intervention.^[12]^

There have been alarming increases in AF-related mortality, with disparities by sex, race, and geography, according to past national analyses.^[13]^ With an increase in the percentage of adults over 65, this age group is most impacted by both AF and NRVHD. Despite these insights, gaps remain. Prior studies have typically focused on AF or valvular heart disease individually. There are limited analyses of their combined burden, leaving unclear how mortality patterns have changed through the 2020s, as the coronavirus disease 2019 pandemic has also interfered with cardiovascular care and likely amplified the risk of thromboembolism through infection-related inflammation and prothrombotic states.^[14]^

Our primary objective was to highlight changes in mortality rates over 25 years and identify disparities within subgroups using the Centers for Disease Control and Prevention (CDC) Wide-ranging Online Data for Epidemiologic Research (WONDER).

## 2. Methods

### 2.1. Study setting and population

We conducted a retrospective analysis of mortality trends involving both AF and NRVHD in the US from 1999 to 2023. Data on mortality trends were extracted from the CDC WONDER database, which provides information on underlying and contributing causes of death, along with basic demographic characteristics from death certificates.^[15]^ The Multiple Cause-of-Death Public Use Record database was utilized to identify cases in which both AF and NRVHD were documented anywhere on the death certificate. This joint identification was chosen because AF and NRVHD frequently coexist and have important clinical and pathophysiological interactions; therefore, analyzing deaths involving both conditions allowed us to specifically assess their combined mortality burden rather than the mortality burden of either condition separately. According to the International Statistical Classification of Diseases and Related Health Problems, 10th Revision(ICD-10), AF was identified using code I48, while NRVHD was identified using codes I34, I35, and I36. The analysis focused on nonrheumatic mitral, aortic, and tricuspid valve disorders (ICD–10 codes I34–I36). I37 (pulmonary valve disorders) was excluded because it accounted for a very small number of deaths and showed incomplete reporting across the study period, including years with insufficient or unavailable mortality data. Given the sparse and inconsistent nature of these data, inclusion of I37 was considered unlikely to provide reliable estimates of long-term mortality trends and could introduce instability into the analysis. Therefore, the study was restricted to I34 to I36, for which mortality data were sufficiently available for longitudinal analysis. This method has been implemented in previous CDC WONDER analyses.^[16,17]^ No institutional review board approval was required for this study, as the analysis was based on de-identified data retrieved from the CDC WONDER database and was conducted in accordance with the Strengthening the Reporting of Observational Studies in Epidemiology guidelines.^[18]^

### 2.2. Outcome definition

The primary outcome was mortality among US decedents in whom both AF and NRVHD were documented anywhere on the death certificate, either as the underlying or contributing cause of death. Mortality trends were assessed using age-adjusted mortality rates (AAMRs) per 100,000 population and annual percent change (APCs) and average annual percentage changes (AAPCs) over the study period.

### 2.3. Data abstraction

The data abstracted in our study were organized according to various demographic factors, including age, sex, race/ethnicity, and location of death. Age groups were divided into 2 categories: 45 to 64 and ≥ 65 years. This categorization was selected to distinguish middle-aged adults from older adults and was consistent with the prespecified study design. Similar age cutoffs have been used in previous studies.^[19,20]^ Ethnicity groups were categorized into Hispanic (Latino) and non-Hispanic (NH), with further subcategorization into White, Black/African American, American Indian/Alaska Native, and Asian. Locations of death included inpatient facilities, outpatient clinics, emergency rooms, sudden deaths, residences, hospice/nursing homes, long-term care facilities, and other unspecified places. Mortality trends were also stratified by geographic Census regions, as defined by the US Census Bureau, by state, and by level of urbanization, categorized as urban metropolitan and rural areas according to the 2013 Urban–Rural Classification Scheme for Counties. Urbanization data were available only through 2020; therefore, analyses stratified by urbanization were restricted to the period 1999 to 2020. All these categorizations have been employed in previous CDC WONDER analyses.^[19–21]^

### 2.4. Statistical analysis

Crude Mortality Rates (CMRs) (deaths per 100,000 person-years) and AAMRs (deaths per 100,000 persons standardized to the 2000 US population) were calculated as per CDC standards for the analysis of the demographic variables related to mortality trends involving both AF and NRVHD. The National Cancer Institute’s Joinpoint Regression Program 5.4.0 was used for the determination of APC values for AAMR in the analysis of temporal trends in AAMR over the study period. Joinpoint regression, a segmented regression technique, identifies points of trend change (join points) by fitting log-linear models and uses permutation tests to select the optimal number of join points (up to 3 allowed), ensuring model fit.^[22]^ APC describes the rate of change of AAMR over time, providing a suitable measure to observe trends in mortality rates, with a positive value showing an increase, whereas a negative value reflects a decrease in mortality rates. The Grid Search Method (0, 2), combined with a permutation test and parametric method, was used to calculate APC values with a 95% confidence interval (CI). A 2tailed *t* test was used to determine the statistical significance of APC values, with *P* < .05 deemed statistically significant.

## 3. Results

Between 1999 and 2023, a total of 141,846 deaths among U.S. adults aged 45 years and older involved both AF and NRVHD. These deaths occurred across various locations, with the majority recorded in medical facilities as inpatients (34.66%), followed by decedents’ homes (29.7%), nursing homes or long-term care settings (21.1%), outpatient or emergency room settings (4.42%), and hospice facilities (5.14%). A smaller proportion of deaths occurred in medical facilities dead on arrival (0.28%), medical facilities status unknown (0.04%), other locations (4.46%), and for 0.10% of cases, the place of death was unknown. The overall AAMR increased from 2.98 in 1999 to 7.3 in 2023. Over the entire study period, the mean AAMR observed from 1999 to 2023 was 4.54 (95% CI: 4.42–4.66), and the AAPC was 4.17 (95% CI: 3.83–4.51). The AAMR increased from 2.98 in 1999 to 3.55 in 2009 with an (APC of 2.51; 95% CI: 0.–3.56), followed by a significant rise to 7.3 in 2023 with an (APC of 5.37; 95% CI: 4.95– 6.04). (Supplemental Tables 1, 2, 3, 4, Supplemental Digital Content 1) (Fig. 1)

**Figure 1.**
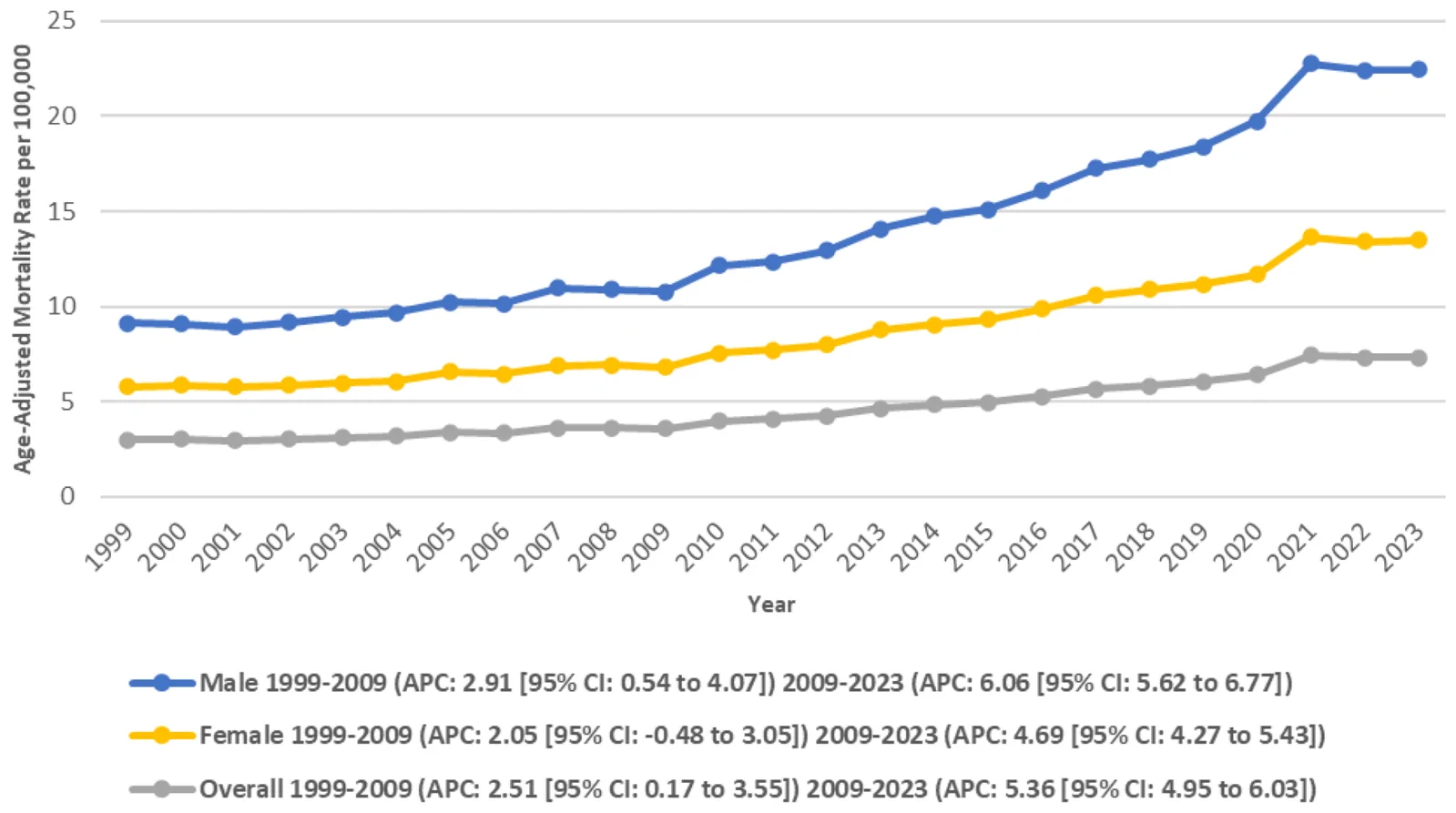
Overall and sex-stratified age-adjusted mortality rates per 100,000 for deaths involving atrial fibrillation and non-rheumatic valvular heart disease among U.S. adults, 1999 to 2023. APC = annual percent change, CI = confidence interval, US = United States.

### 3.1. Stratified by sex

Across the duration of the study period from 1999 to 2023, there were 63,901 deaths among males and 77,945 deaths among females. Notably, males exhibited a higher overall AAMR compared to women (overall AAMR for men: 5.31; 95%CI: 5.09 to 5.52; for women: 4.02; 95%CI: 3.87 to 4.12). On average, the AAMR for both males and females changed during the study period, with males exhibiting a significantly greater increasing trend than females (AAPC for men: 4.74; 95% CI: 4.34–5.12; AAPC for females: 3.59; 95% CI: 3.26–3.92).

The AAMR for males increased gradually from 3.32 in 1999 to 3.97 in 2009 with an (APC of 2.92; 95% CI: 0.55–4.07), followed by a significant increasing trend from 3.97 in 2009 to 8.96 in 2023 with an (APC of 6.06; 95% CI: 5.62–6.78). In contrast, the trend for AAMR among females also showed a similar pattern but was less significant than males, with an AAMR increasing from 2.8 in 1999 to 3.26 in 2009 with an (APC of 2.05; 95% CI: −0.49–3.05), followed by a considerable surge from 3.26 in 2009 to 6.18 in 2023 with an (APC of 4.69; 95% CI: 4.28– 5.43). (Supplemental Tables 1, 3, 4, Supplemental Digital Content 1) (Fig. 1)

### 3.2. Stratified by race/ethnicity

Marked heterogeneities in AAMR were observed among different racial groups. The highest AAMR was recorded among NH Whites, with noticeable differences across other groups, including NH Black or African Americans, Hispanics, and NH Asian or Pacific Islanders (overall AAMR: NH Whites: 5.17; 95%CI: 5.03–5.31; NH Asian or Pacific Islanders: 1.89; 95% CI: 1.48 to 2.37; Hispanics: 1.88; 95% CI: 1.58–2.21; NH Black or African Americans: 1.63; 95% CI: 1.38–1.89).

The AAMR of all racial groups exhibited distinct trends from 1999 to 2023, with the incline most pronounced in NH Whites (AAPC: 4.51; 95% CI: 4.19–4.82); NH Black or African Americans (AAPC: 4.28; 95% CI: 3.62–5.27); NH Asian or Pacific Islanders (AAPC: 3.47; 95% CI: 2.79–4.65); and Hispanics (AAPC: 3.46; 95% CI: 2.28–5.41).

Among NH Whites, the AAMR increased moderately from 3.23 in 1999 to 3.94 in 2009 with an (APC of 2.71; 95% CI: 0.85–3.67), followed by a significant rise from 3.94 in 2009 to 8.65 in 2023 with an (APC of 5.82; 95% CI: 5.40–6.46). For NH Black or African Americans, the AAMR showed a different trend with an initial decrease from 1.16 in 1999 to 1.04 in 2007 with an (APC of −0.42; 95% CI: −10.15–3.23), followed by a rising trend from 1.04 in 2007 to 2.88 in 2023 with an (APC of 6.72; 95% CI: 5.95–8.39). For NH Asian or Pacific Islanders, there was an overall increase in AAMR from 1.65 in 1999 to 2.91 in 2023 with an (APC of 3.47; 95% CI: 2.79–4.65) with no joinpoints identified. For the Hispanic population, the AAMR showed an initial decrease from 1.47 in 1999 to 0.93 in 2004 with an (APC of −4.84; 95% CI: −23.65–4.95), followed by a significant rise from 0.93 in 2004 to 3.07 in 2023 with an (APC of 5.77; 95% CI: 4.80–9.89). (Supplemental Table 1, 3, 5, Supplemental Digital Content 1) (Fig. 2)

**Figure 2.**
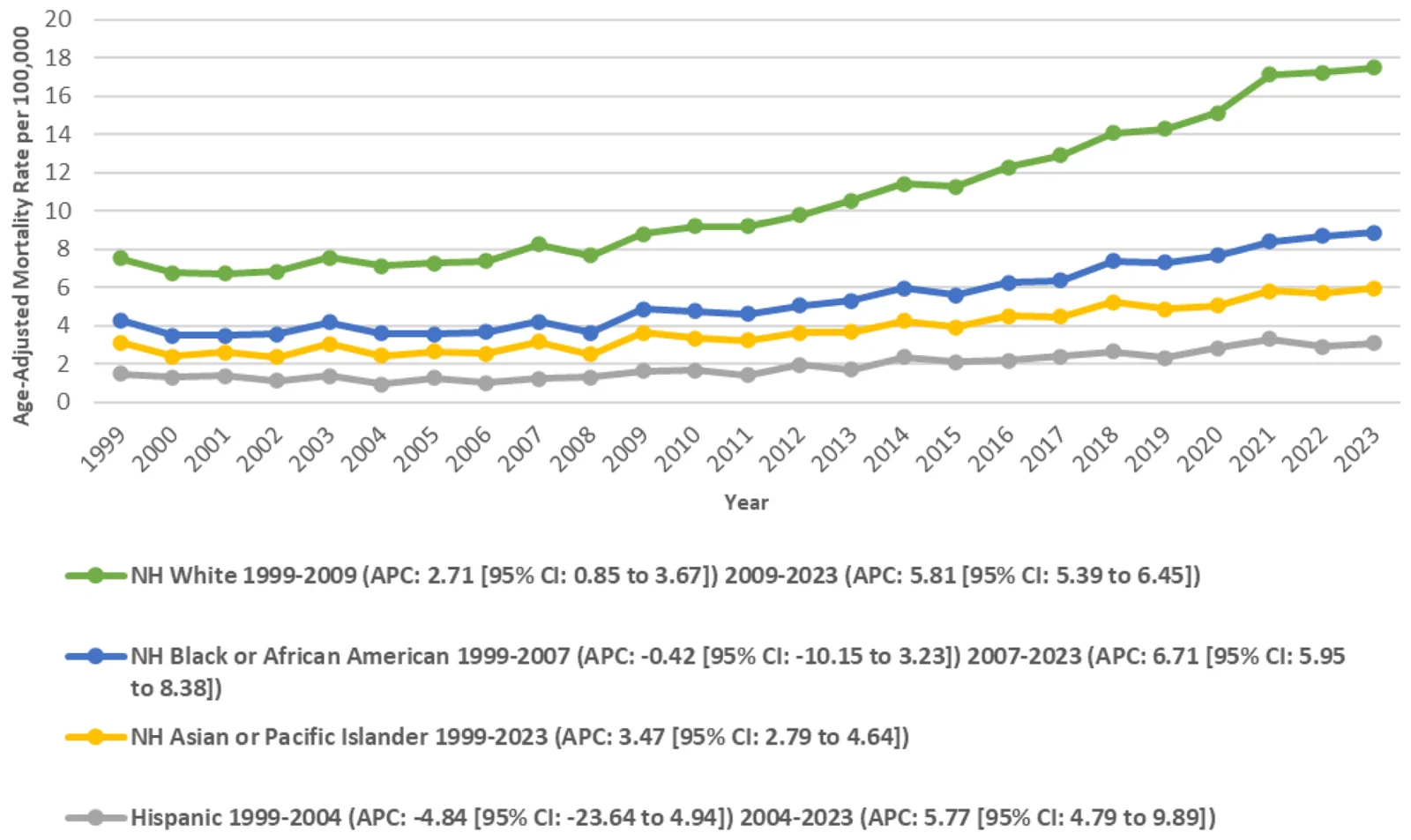
age-adjusted mortality rates per 100,000 for deaths involving atrial fibrillation and non-rheumatic valvular heart disease, stratified by race, among U.S. adults, 1999 to 2023. APC = annual percent change, CI = confidence interval, US = United States.

### 3.3. Stratified by census region

On average, over the study period, the highest mortality was observed in the West region (AAMR: 5.73; 95% CI: 5.44–6.02), followed by the Northeast (AAMR: 4.82; 95% CI: 4.54–5.09), Midwest (AAMR: 4.79; 95% CI: 4.54–5.05), and South region (AAMR: 3.48; 95% CI: 3.31–3.66). The AAMR of all 4 regions changed from 1999 to 2023, with the greatest incline observed in the South with an (AAPC of 4.46; 95% CI: 4.10–4.87), Midwest (AAPC: 4.17; 95% CI: 3.81–4.57), West (AAPC: 3.69; 95%CI: 3.2–4.17), and Northeast (AAPC: 3.64; 95% CI: 3.25–4.05).

The West region showed an initial decrease in AAMR from 3.82 in 1999 to 3.69 in 2002 with an (APC of −1.56; 95% CI: −8.74–3.45), followed by a significant rise from 3.69 in 2002 to 8.72 in 2023 with an (APC of 4.46; 95% CI: 4.24–4.89). For the Northeast region, the AAMR showed a gradual increase from 3.47 in 1999 to 3.94 in 2009 with an (APC of 1.46; 95% CI: −0.802.66), accompanied by a significant rise from 3.94 in 2009 to 7.75 in 2023 with an (APC of 5.23; 95% CI: 4.69–6.07). For the Midwest, there was a gradual increase from 3.04 in 1999 to 3.48 in 2009 with an (APC of 2.25; 95% CI: 0.34–3.44), followed by a notable rise from 3.48 in 2009 to 8.12 in 2021 with an (APC of 6.54; 95% CI: 5.9–9.32). Afterwards, there was a gentler decrease from 8.12 in 2021 to 7.89 in 2023 with an (APC of −0.12; 95% CI: −4.03–4.92). The South region showed an increase in AAMR from 2.18 in 1999 to 2.73 in 2011 with an (APC of 2.82; 95% CI: 0.76–3.79), followed by a remarkable increase from 2.73 in 2011 to 5.89 in 2023 with an (APC of 6.13; 95% CI: 5.47–7.25). (Supplemental Table 3, 6, Supplemental Digital Content 2) (Fig. 3).

**Figure 3.**
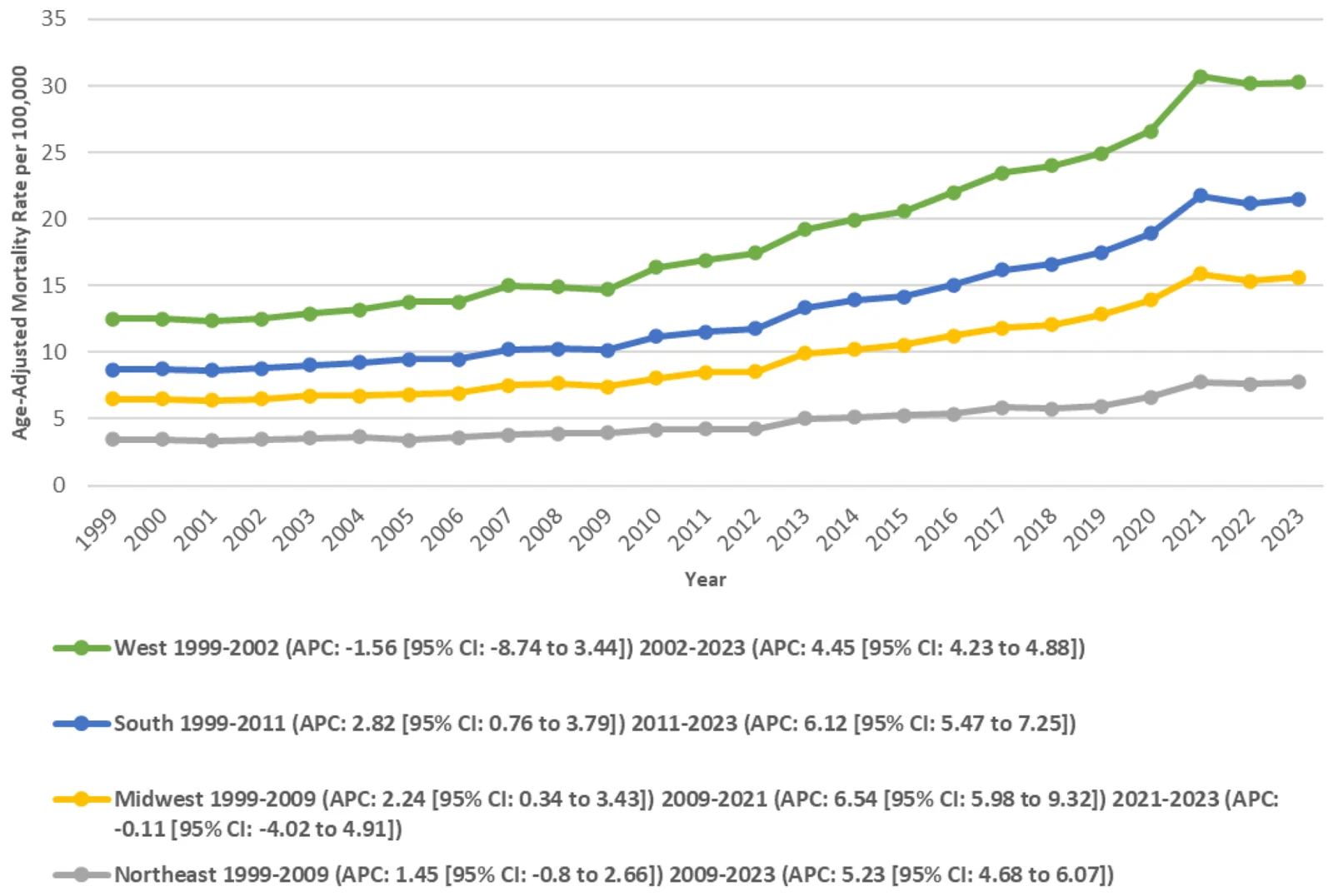
age-adjusted mortality rates per 100,000 for deaths involving atrial fibrillation and non-rheumatic valvular heart disease, stratified by census region, among U.S. adults, 1999 to 2023. APC = annual percent change, CI = confidence interval, US = United States.

### 3.4. Stratified by urbanization

Both nonmetropolitan (rural) and metropolitan (urban) areas exhibited differing trends in AAMR throughout the study period from 1999 to 2020. The overall AAMR was significantly higher and showed a steeper incline in nonmetropolitan areas (AAMR: 4.36; 95% CI: 4.08–4.64) compared to metropolitan areas (AAMR: 4.10; 95% CI: 3.97–4.23). The AAPC for nonmetropolitan areas was (4.61; 95% CI: 4.15–5.08), and for metropolitan areas (AAPC: 3.49; 95% CI: 3.29–3.92).

The AAMR for nonmetropolitan areas showed an increasing trend from 2.81 in 1999 to 4.04 in 2011 with an (APC of 3.26; 95% CI: 1.15–4.18), and continued to increase until 2020 with an AAMR of 7.31 with (APC of 6.43; 95% CI: 5.44–8.70). For metropolitan areas, the AAMR initially decreased from 3.01 in 1999 to 2.88 in 2001 with an (APC of −1.97; 95% CI: −4.77– 2.69), followed by a gentler rise from 2.88 in 2001 to 3.52 in 2009 with an (APC of 2.85; 95% CI: 1.75–6.84), superseded by a significant rise from 3.52 in 2009 to 6.2 in 2020 with an (APC of 4.99; 95% CI: 3.54–6.54). (Supplemental Table 3, 7, Supplemental Digital Content 2) (Fig. 4).

**Figure 4.**
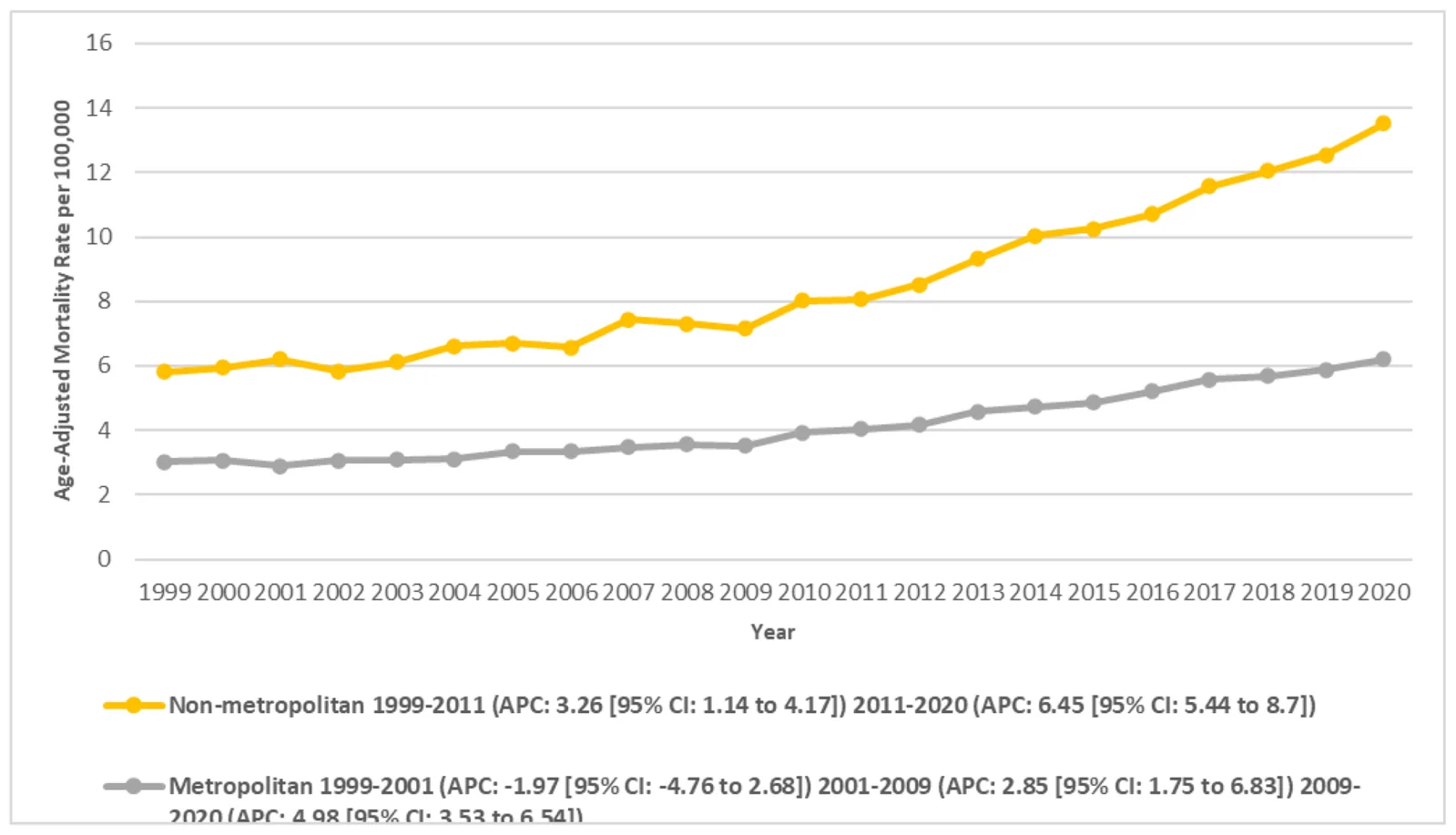
age-adjusted mortality rates per 100,000 for deaths involving atrial fibrillation and non-rheumatic valvular heart disease, stratified by urban-rural classification, among U.S. adults, 1999 to 2020. APC = annual percent change, CI = confidence interval, US = United States.

### 3.5. Stratified by age

Across the duration of the study period, older adults aged 65+ years showed significantly higher CMR compared to young and middle-aged groups (overall CMR for 65+ years: 12.17; 95% CI: 11.84–12.49; for age group 45 to 64 years, overall CMR: 0.20; 95% CI: 0.17–0.23). On average, the CMR of both age groups changed from 1999 to 2023, with the greatest magnitude of increase observed among adults aged 45 to 64 years (AAPC: 6.28; 95% CI: 5.45–7.26) and for age group 65 + years (AAPC: 3.38; 95% CI: 3.14–3.69).

The CMR for adults aged 65+ years showed a variable trend with an initial gradual increase from 8.01 in 1999 to 8.35 in 2003 (with an APC of 1.32; 95% CI: −2.91–3.60), followed by a significant rise to 17.48 in 2021 (with an APC of 4.09; 95% CI: 3.96–6.27). Afterward, there was a gentler decrease from 17.48 in 2021 to 17.22 in 2023 (with an APC of 1.14; 95% CI: −1.07– 3.77). For adults aged 45 to 64 years, the CMR showed both increasing and decreasing trends, with an initial increase from 0.12 in 1999 to 0.18 in 2008 and then decreasing from 0.18 in 2008 to 0.12 in 2011 (with an APC of 3.08; 95% CI: −3.09–5.47), followed by an inclining trend from 0.12 in 2011 to 0.34 in 2023 (with an APC of 9.57; 95% CI: 7.83–14.20). (Supplemental Table 3, 8, Supplemental Digital Content 2) (Fig. 5).

**Figure 5.**
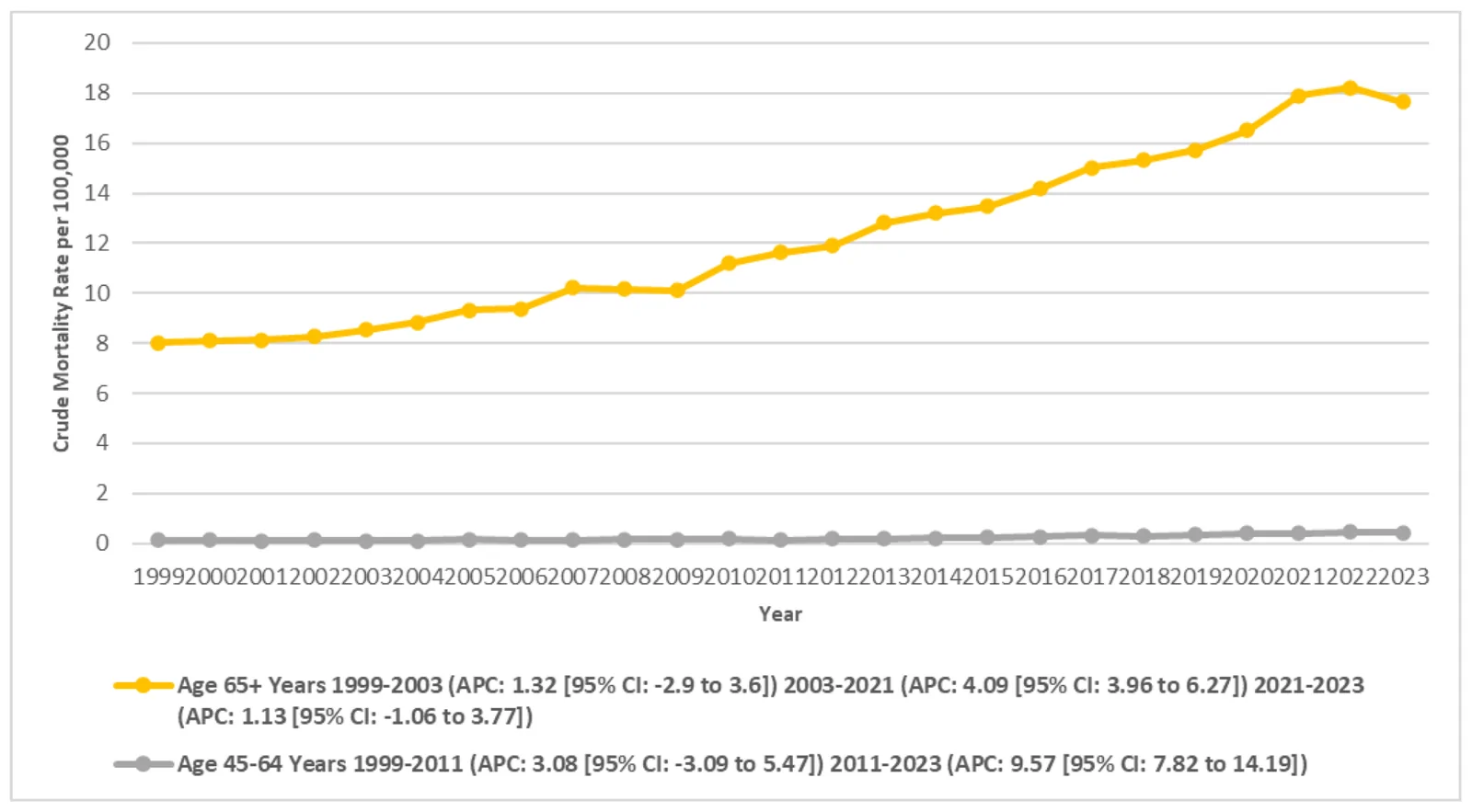
Crude mortality rates per 100,000 for deaths involving atrial fibrillation and non-rheumatic valvular heart disease, stratified by age group, asmong U.S. adults, 1999 to 2023. APC = annual percent change, CI = confidence interval, US = United States.

### 3.6. Stratified by state

From 1999 to 2020, the highest AAMR was observed in Vermont (AAMR: 11.94; 95% CI: 11.– 12.83) and the lowest AAMR was reported in Georgia (AAMR: 1.87; 95% CI: 1.76–1.98). States falling within the top 90^th^ percentile included Alaska, Delaware, Iowa, Maine, Minnesota, Oregon, and California. Whereas, the states falling within the bottom 10^th^ percentile included Arizona, Arkansas, District of Columbia, Florida, New Mexico, Illinois, Kansas, Kentucky, Louisiana, Michigan, Mississippi, New York, Utah, and West Virginia.

From 2021 to 2023, variations in AAMR were observed among different states. State-level AAMRs ranged from 2.62 (95% CI: 2.–2.93) in Georgia to 21.71 (95% CI: 20.47–22.95) in Oregon. States falling within the top 90th percentile included Alaska, California, Colorado, Idaho, Iowa, Maine, Maryland, Minnesota, Nebraska, New Hampshire, Pennsylvania, South Dakota, Washington, Wisconsin, and Wyoming. Whereas, states falling within the bottom 10th percentile included Arkansas, Connecticut, District of Columbia, Georgia, Illinois, Louisiana, Michigan, New Mexico, and Utah (Supplemental Table 9, 10, Supplemental Digital Content 3).

## 4. Discussion

We observed a steady increase in mortality involving both AF and NRVHD from 1999 to 2023 among adults aged+ 45, with AAMRs rising from 2.98 to 7.30 over the study period. Males consistently experienced higher mortality than females, and their upward trend was steeper. Among racial and ethnic groups, NH White patients carried the highest mortality burden, followed by NH Black, Hispanic, and NH Asian or Pacific Islander populations. Age-specific analyses showed that adults aged 65 years and older accounted for the greatest mortality, although the most pronounced relative increase occurred in those aged 45 to 64 years. Geographically, the Western region exhibited the highest mortality, while the South reported the lowest, though upward trends were observed across all regions. Rural areas bore a higher mortality burden compared with urban areas. At the state level, Vermont and Oregon reported the highest mortality in different periods from 1999 to 2020 and 2021 to 2023, respectively, while Georgia and Alabama had the lowest.

Emerging evidence highlights a complex interplay between AF and NRVHD, particularly mitral regurgitation, in determining clinical outcomes. Structural changes in the atrium and valves, including annular dilatation, atrial enlargement, and fibrosis, may create a substrate for AF, while AF itself worsens valve dysfunction through atrial dilatation and functional regurgitation.^[23]^ Large cohort studies, such as the Impact of Valvular Heart Disease on Oral Anticoagulant Therapy in Non-Valvular Atrial Fibrillation Study trial, demonstrate that many patients classified as having non-valvular AF in fact harbor significant valve disease, most commonly mitral regurgitation. These patients face higher rates of stroke, bleeding, and adverse cardiovascular events compared with those without NRVHD.^[24]^ Population-based analyses further indicate that both valvular AF and non-valvular AF with coexisting valve pathology increase risks of mortality and heart failure hospitalizations. However, adjustment for echocardiographic markers of remodeling attenuates these associations, suggesting that structural disease burden, rather than AF type alone, drives poor outcomes.^[25]^

AAMRs for AF and NRVHD rose steadily from 1999 to 2009, then accelerated through 2023. This rise may reflect multiple factors: a growing prevalence of obesity, diabetes, hypertension, dyslipidemia, and chronic obstructive pulmonary disease, combined with an aging population that increased absolute death counts, particularly among older adults. Disparities in diagnosis, treatment, and healthcare access across sex, race, and geography may have contributed to the observed mortality differences, particularly among men, NH White individuals, and rural populations. Changes in ICD coding practices, improved screening, and more complete death-certificate documentation may also have influenced the reporting of AF and NRVHD over time. Despite therapeutic advances, including the use of direct oral anticoagulants, mortality involving AF and NRVHD remained substantial during the study period. The persistent mortality burden may reflect the coexistence of coronary artery disease and heart failure, while COVID-19-related systemic inflammation, prothrombotic states, and myocardial injury may have contributed to changes in cardiovascular outcomes during the pandemic. Unfavorable lifestyle patterns may also have played a role.^[24,26]^

Research on tricuspid valve disease has shown a marked rise in mortality from NRVHD after 2012, particularly among patients with tricuspid regurgitation. This increase may be related to an aging population with multiple comorbidities, a higher prevalence of heart failure, increased pacemaker implantation, cor pulmonale, and left ventricular assist device use, which may worsen tricuspid regurgitation through mechanisms such as interventricular septal shift.^[27]^

Males consistently showed higher AAMRs than females, with a sharper rise from 2009 onward. This disparity may reflect differences in the prevalence of cardiovascular comorbidities and risk factors, including coronary artery disease, obesity, heart failure, diabetes, alcohol use, peripheral vascular disease, and renal disease, as well as differences in the clinical management of AF and valvular heart disease.^[17,28,29]^ Differences in diagnosis, healthcare utilization, and referral for valvular interventions may also contribute to the observed sex disparity. Females had lower overall mortality and a less pronounced increase over time. This disparity may partly reflect sex-related biological differences, including potential effects of estrogen on atrial remodeling, nitric oxide activity, and endothelial function.^[28,30]^ In valvular disease, differences in cardiovascular risk profiles and longevity may also contribute to the observed mortality patterns.^[17]^

NH White individuals experienced a higher AAMR involving both AF and NRVHD compared with African Americans and other racial groups between 1999 and 2023. Structural factors such as larger left atrial size, genetic traits associated with AF, and a higher proportion of sustained AF may contribute to cumulative risk.^[31–33]^ Whites also demonstrate the “AF race paradox” with higher AF incidence and mortality despite lower prevalence of some cardiovascular risk factors.^[32]^ Other factors that may contribute to this disparity include differences in healthcare access, routine cardiac evaluation, specialist care, age distribution, survival, and the prevalence of comorbidities such as hypertension, diabetes, obesity, and coronary artery disease.^[17,32,34]^ In contrast, African Americans and other minority populations show lower observed incidence and mortality involving both AF and NRVHD. Higher overall and cardiovascular mortality in these groups may reduce survival to the ages at which AF commonly develops, while differences in AF presentation, including a greater proportion of paroxysmal AF, may contribute to differences in detected incidence.^[31,35]^ Structural and genetic differences, including differences in left atrial size and the prevalence of high-risk paired like homeodomain 2 (gene)variants, may also influence susceptibility to AF. For NRVHD, differences in diagnosis, referral patterns, access to cardiac imaging, financial barriers, and healthcare utilization may contribute to the lower observed mortality, although an underlying burden of disease may remain underrecognized.^[17,35]^

Adults aged 65 years and older consistently had the highest CMR. The higher mortality observed in older adults may reflect age-related atrial remodeling, left atrial enlargement, fibrosis, amyloid deposition, reduced atrial appendage flow, and age-related valvular abnormalities, which may increase susceptibility to AF, thromboembolic complications, and stroke.^[17,36]^ Comorbidities, including heart failure, coronary artery disease, hypertension, diabetes, obesity, metabolic syndrome, and sleep apnea, may further contribute to this increased mortality burden.^[17,36]^ The higher mortality among older adults may also reflect cumulative valvular degeneration and comorbidity burden, whereas younger adults may benefit from greater access to contemporary surgical, percutaneous, and medical interventions.^[17,37]^ Aging also may accelerate AF progression through atrial fibrosis, cardiomyocyte hypertrophy, conduction slowing, and ionic current alterations. Age-related arterial stiffness and diastolic dysfunction may increase atrial pressures and remodeling, predisposing older individuals to transition from paroxysmal to persistent AF, particularly with structural heart disease.^[38,39]^ The Western U.S. consistently shows the highest CMR, influenced by a larger older adult population, higher comorbidity prevalence, and interactions between AF and heart failure.^[17]^

Regional healthcare disparities, especially in rural and nonmetropolitan areas, may limit access to specialists, direct oral anticoagulants, rhythm control, and cardiac rehabilitation. Provider shortages, reliance on urgent care, financial barriers, and lower awareness further worsen outcomes.^[17,35]^ Improved diagnostic practices and death-certificate reporting may have contributed to changes in recorded mortality, while COVID-19–related cardiovascular complications may also have influenced mortality patterns during the pandemic, despite advances in treatment.^[17,35]^ Rural areas consistently experienced higher mortality than urban regions. This disparity may reflect differences in access to specialty and primary care, cardiology and electrophysiology services, healthcare infrastructure, hospital availability, and emergency medical services, which may contribute to delays in diagnosis and treatment.^[17,34,35]^ Lower socioeconomic status, restrictive Medicaid policies, financial barriers, and reduced self-management of AF may also contribute. Rural populations may also have higher burdens of smoking, hypertension, diabetes, obesity, and other cardiovascular comorbidities, along with differences in the use of direct oral anticoagulants, rhythm-control strategies, and cardiac rehabilitation, which may contribute to the observed mortality disparity.^[17,35]^ These disparities may contribute to the higher mortality observed in rural populations compared with their urban counterparts and may reflect differences in access to advanced AF therapies, such as electrical cardioversion and catheter ablation.^[40,41]^

## 5. Limitations

Our study has several limitations. First, the analysis relied on (ICD-10) codes assigned on death certificates, which may be subject to misclassification or incomplete documentation. This may limit the clinical detail available and affect the accuracy of mortality estimates. Additionally, information on socioeconomic status, health insurance, education, and occupation was unavailable, limiting adjustment for potential social and socioeconomic determinants of mortality.^[42–44]^ The database also lacks patient-level clinical and laboratory information, including valvular disease phenotype, imaging findings, genetic markers, treatment history, and key comorbidities such as hypertension, coronary artery disease, heart failure, diabetes, chronic kidney disease, obesity, and smoking. Consequently, assessment of disease severity, treatment effects, and adjustment for these potential confounders were limited.^[42,45]^ Timing of interventions and adherence to therapy were also unavailable, precluding assessment of how delays in care may have affected outcomes.^[46]^ The broad ≥ 65-year age category may have limited our ability to identify differences in mortality patterns among specific older age groups. Additionally, pulmonary valve disorders ICD-10 code I37 were not included because of their very low number of reported deaths and incomplete reporting across the study period, which limited the reliability and stability of long-term trend estimates. Therefore, our findings may not be generalizable to mortality involving nonrheumatic pulmonary valve disorders.

Finally, the retrospective nature of the study, along with changes in diagnostic practices and increasing awareness and recognition of AF and NRVHD, may have influenced observed mortality trends. Improvements in documentation and reporting may also have contributed to changes in recorded mortality over time.^[45]^ Future studies incorporating detailed clinical, treatment, and socioeconomic information are warranted to further characterize mortality trends and disparities involving AF and NRVHD.

## 6. Conclusion

Mortality involving AF and NRVHD steadily increased, with an accelerated rise in recent years. This trend is multifactorial, driven by aging, rising prevalence of cardiovascular comorbidities, and unfavorable lifestyle patterns. Mortality disparities exist across sex, race, age, and geography. Males, NH White individuals, older adults, and residents of rural or Western regions consistently show higher mortality, reflecting structural heart differences, comorbidity burden, limited healthcare access, and underutilized interventions. AF and valvular disease interact synergistically, with structural remodeling and valve dysfunction amplifying adverse outcomes. Despite advances in therapy, including anticoagulation and rhythm-control strategies, these trends highlight persistent gaps in prevention, diagnosis, and management. Addressing disparities and targeting high-risk populations is essential to reducing mortality involving AF and NRVHD.

## Author contributions

**Conceptualization:** Ibtissam ElGhazzani, Mohamed Fawzi Hemida.

**Data curation:** Ibtissam ElGhazzani, Youssef Ramadan, Muhammad Sarim Azad Khan, Krish Patel, FNU Sahil.

**Formal analysis:** Ibtissam ElGhazzani, Youssef Ramadan, Alyaa Ahmed Ibrahim, Maryam Saghir, Abdul Samad, Eshal Saghir, FNU Sahil, Maha Sajjad.

**Methodology:** Ibtissam ElGhazzani.

**Investigation:** Alyaa Ahmed Ibrahim, Maryam Saghir, Abdul Samad, Eshal Saghir, FNU Sahil.

**Validation:** Muhammad Sarim Azad Khan, Krish Patel, Maha Sajjad, Yahya Samadi.

**Visualization:** Muhammad Sarim Azad Khan, Krish Patel, Maha Sajjad, Yahya Samadi.

**Resources:** Karim Othman Abdelnaim, Moustafa Ahmed Elsayed.

**Project administration:** Mohamed Fawzi Hemida.

**Supervision:** Mohamed Fawzi Hemida.

**Writing – original draft:** Ibtissam ElGhazzani, Youssef Ramadan, Alyaa Ahmed Ibrahim, Maryam Saghir, Abdul Samad, Amr Arafa, Krish Patel, Eshal Saghir, FNU Sahil, Maha Sajjad, Yahya Samadi.

**Writing – review & editing:** Ibtissam ElGhazzani, Youssef Ramadan, Alyaa Ahmed Ibrahim, Maryam Saghir, Muhammad Sarim Azad Khan, Karim Othman Abdelnaim, Moustafa Ahmed Elsayed, Abdul Samad, Amr Arafa, Krish Patel, Eshal Saghir, FNU Sahil, Maha Sajjad, Yahya Samadi, Mohamed Fawzi Hemida.




















