# Supplementary material for: Mortality involving non-rheumatic valvular heart disease and atrial fibrillation in the United States: Long-term trends and implications
Source: Medicine (Baltimore). 2026 Oct 9;105(41):e51020. doi: 10.1097/MD.0000000000051020 (PMC13657543; doi:10.1097/MD.0000000000051020)
Supplement: Supplementary file 1 [file medi-105-e51020-s001.docx]

**Supplemental Table 1** Atrial Fibrillation and Non-Rheumatic Valvular Heart Disease–related Deaths, Stratified by Sex and Race, in Adults in the United States, 1999 to 2023

| **Year** | **Overall** | **Women** | **Men** | **NH White** | **NH Black or African American** | **NH Asian or Pacific Islander** | **NH American Indian or Alaska Native** | **Hispanic or Latino** |
| --- | --- | --- | --- | --- | --- | --- | --- | --- |
| 1999 | 2,823 | 1,716 | 1,107 | 2,645 | 83 | 32 | Missing | 55 |
| 2000 | 2,872 | 1,798 | 1,074 | 2,711 | 80 | 22 | Missing | 56 |
| 2001 | 2,894 | 1,808 | 1,086 | 2,741 | 67 | 24 | Missing | 58 |
| 2002 | 2,978 | 1,817 | 1,161 | 2,808 | 91 | 27 | Missing | 48 |
| 2003 | 3,099 | 1,871 | 1,228 | 2,909 | 83 | 38 | Missing | 63 |
| 2004 | 3,242 | 1,934 | 1,308 | 3,058 | 93 | 35 | Missing | 49 |
| 2005 | 3,465 | 2,121 | 1,344 | 3,279 | 77 | 38 | Missing | 67 |
| 2006 | 3,539 | 2,112 | 1,427 | 3,347 | 90 | 44 | Missing | 53 |
| 2007 | 3,916 | 2,300 | 1,616 | 3,691 | 88 | 58 | Missing | 71 |
| 2008 | 3,994 | 2,382 | 1,612 | 3,771 | 102 | 35 | Missing | 80 |
| 2009 | 4,058 | 2,383 | 1,675 | 3,763 | 111 | 68 | Missing | 108 |
| 2010 | 4,581 | 2,606 | 1,975 | 4,266 | 129 | 60 | 12 | 114 |
| 2011 | 4,870 | 2,781 | 2,089 | 4,549 | 132 | 71 | 13 | 105 |
| 2012 | 5,207 | 2,934 | 2,273 | 4,827 | 139 | 70 | 16 | 155 |
| 2013 | 5,801 | 3,281 | 2,520 | 5,379 | 173 | 89 | 14 | 146 |
| 2014 | 6,187 | 3,387 | 2,800 | 5,675 | 191 | 93 | 16 | 212 |
| 2015 | 6,507 | 3,587 | 2,920 | 6,004 | 183 | 96 | 20 | 204 |
| 2016 | 7,073 | 3,831 | 3,242 | 6,498 | 202 | 129 | 19 | 225 |
| 2017 | 7,751 | 4,175 | 3,576 | 7,117 | 231 | 124 | 21 | 258 |
| 2018 | 8,130 | 4,357 | 3,773 | 7,390 | 267 | 161 | 17 | 295 |
| 2019 | 8,589 | 4,474 | 4,115 | 7,808 | 306 | 169 | 30 | 276 |
| 2020 | 9,302 | 4,635 | 4,667 | 8,422 | 350 | 159 | 28 | 343 |
| 2021 | 9,996 | 5,045 | 4,951 | 9,063 | 322 | 168 | 41 | 398 |
| 2022 | 10,531 | 5,363 | 5,168 | 9,525 | 392 | 204 | 36 | 363 |
| 2023 | 10,441 | 5,247 | 5,194 | 9,409 | 385 | 216 | 28 | 392 |
| **Total** | 141,846 | 77,945 | 63,901 | 130,655 | 4,367 | 2,230 | 311 | 4,194 |

NH, non-Hispanic
