# Supplementary material for: Mortality involving non-rheumatic valvular heart disease and atrial fibrillation in the United States: Long-term trends and implications
Source: Medicine (Baltimore). 2026 Oct 9;105(41):e51020. doi: 10.1097/MD.0000000000051020 (PMC13657543; doi:10.1097/MD.0000000000051020)
Supplement: Supplementary file 2 [file medi-105-e51020-s002.docx]

**Supplemental Table 3** Annual percent change (APC) of Atrial Fibrillation and Non-Rheumatic Valvular Heart Disease–related Age-Adjusted Mortality Rates per 100,000 in Adults in the United States, 1999 to 2023

| **Year Interval** | **APC (95% CI)** |
| --- | --- |
| **Overall** |  |
| 1999-2009 | 2.51 (0.17 to 3.55) |
| 2009-2023 | 5.36 (4.95 to 6.03) |
| **Women** |  |
| 1999-2009 | 2.05 (−0.48 to 3.05) |
| 2009-2023 | 4.69 (4.27 to 5.43) |
| **Men** |  |
| 1999-2009 | 2.91 (0.54 to 4.07) |
| 2009-2023 | 6.06 (5.62 to 6.77) |
| **NH White** |  |
| 1999-2009 | 2.71 (0.85 to 3.67) |
| 2009-2023 | 5.81 (5.39 to 6.45) |
| **NH Black or African American** | |
| 1999-2007 | −0.42 (−10.15 to 3.23) |
| 2007-2023 | 6.71 (5.95 to 8.38) |
| **NH Asian or Pacific Islander** |  |
| 1999-2023 | 3.47 (2.79 to 4.64) |
| **Hispanic or Latino** |  |
| 1999-2004 | −4.84 (−23.64 to 4.94) |
| 2004-2023 | 5.77 (4.79 to 9.89) |
| **Metropolitan** |  |
| 1999-2001 | −1.97 (−4.76 to 2.68) |
| 2001-2009 | 2.85 (1.75 to 6.83) |
| 2009-2020 | 4.98 (3.53 to 6.54) |
| **Non-metropolitan** |  |
| 1999-2011 | 3.63 (1.14 to 4.17) |
| 2011-2020 | 6.42 (5.44 to 8.7) |
| **Northeast** |  |
| 1999-2009 | 1.45 (−0.8 to 2.66) |
| 2009-2023 | 5.23 (4.68 to 6.07) |
| **Midwest** |  |
| 1999-2009 | 2.24 (0.34 to 3.43) |
| 2009-2021 | 6.54 (5.98 to 9.32) |
| 2021-2023 | −0.11 (−4.02 to 4.91) |
| **South** |  |
| 1999-2011 | 2.82 (0.76 to 3.79) |
| 2011-2023 | 6.12 (5.47 to 7.25) |
| **West** |  |
| 1999-2002 | −1.56 (−8.74 to 3.44) |
| 2002-2023 | 4.45 (4.23 to 4.88) |
| **Age 45-64 Years** |  |
| 1999-2011 | 3.08 (−3.09 to 5.47) |
| 2011-2023 | 9.57 (7.82 to 14.19) |
| **Age 65+ Years** |  |
| 1999-2003 | 1.32 (−2.9 to 3.6) |
| 2003-2021 | 4.09 (3.96 to 6.27) |
| 2021-2023 | 1.13 (−1.06 to 3.77) |

APC = annual percent change; NH = non-Hispanic
