# Supplementary material for: Mortality involving non-rheumatic valvular heart disease and atrial fibrillation in the United States: Long-term trends and implications
Source: Medicine (Baltimore). 2026 Oct 9;105(41):e51020. doi: 10.1097/MD.0000000000051020 (PMC13657543; doi:10.1097/MD.0000000000051020)
Supplement: Supplementary file 3 [file medi-105-e51020-s003.docx]

**Supplemental Table 9** Atrial Fibrillation and Non-Rheumatic Valvular Heart Disease–related Age-Adjusted Mortality Rates per 100,000, Stratified by States in Adults in the United States, 1999 to 2020.

| **State** | **Age Adjusted Rate** |
| --- | --- |
| Alabama | 2.5 (2.35-2.66) |
| Alaska | 5.95 (5.01-6.89) |
| Arizona | 2.78 (2.64-2.92) |
| Arkansas | 2.51 (2.31-2.71) |
| California | 5.42 (5.33-5.51) |
| Colorado | 4.88 (4.64-5.12) |
| Connecticut | 4.07 (3.86-4.29) |
| Delaware | 5.04 (4.54-5.55) |
| District of Columbia | 2.55 (2.09-3.01) |
| Florida | 2.71 (2.64-2.78) |
| Georgia | 1.87 (1.76-1.98) |
| Hawaii | 3.83 (3.49-4.16) |
| Idaho | 6 (5.56-6.45) |
| Illinois | 3.06 (2.96-3.17) |
| Indiana | 4.49 (4.31-4.67) |
| Iowa | 5.63 (5.37-5.89) |
| Kansas | 3.33 (3.11-3.56) |
| Kentucky | 3.4 (3.21-3.6) |
| Louisiana | 1.87 (1.72-2.01) |
| Maine | 6.04 (5.62-6.46) |
| Maryland | 4.98 (4.77-5.19) |
| Massachusetts | 4.37 (4.21-4.54) |
| Michigan | 3.24 (3.12-3.36) |
| Minnesota | 7.9 (7.65-8.15) |
| Mississippi | 2.58 (2.37-2.79) |
| Missouri | 3.84 (3.67-4.01) |
| Montana | 4.76 (4.32-5.21) |
| Nebraska | 5.41 (5.06-5.75) |
| Nevada | 2.35 (2.12-2.59) |
| New Hampshire | 6.9 (6.41-7.38) |
| New Jersey | 4.85 (4.7-5) |
| New Mexico | 2.29 (2.06-2.53) |
| New York | 2.74 (2.66-2.82) |
| North Carolina | 4.13 (3.98-4.27) |
| North Dakota | 4.93 (4.41-5.45) |
| Ohio | 4.99 (4.86-5.13) |
| Oklahoma | 3.26 (3.06-3.46) |
| Oregon | 10.24 (9.9-10.57) |
| Pennsylvania | 6.09 (5.95-6.22) |
| Rhode Island | 5.79 (5.34-6.25) |
| South Carolina | 4.34 (4.13-4.56) |
| South Dakota | 4.58 (4.11-5.04) |
| Tennessee | 4.05 (3.88-4.23) |
| Texas | 3.75 (3.65-3.84) |
| Utah | 3.42 (3.12-3.71) |
| Vermont | 11.94 (11.05-12.83) |
| Virginia | 3.57 (3.41-3.72) |
| Washington | 9.51 (9.25-9.77) |
| West Virginia | 3.9 (3.61-4.19) |
| Wisconsin | 5.56 (5.36-5.77) |
| Wyoming | 4.56 (3.91-5.21) |
