# Supplementary material for: Mortality involving non-rheumatic valvular heart disease and atrial fibrillation in the United States: Long-term trends and implications
Source: Medicine (Baltimore). 2026 Oct 9;105(41):e51020. doi: 10.1097/MD.0000000000051020 (PMC13657543; doi:10.1097/MD.0000000000051020)
Supplement: Supplementary file 4 [file medi-105-e51020-s004.docx]

**Supplemental Table 2** Atrial Fibrillation and Non-Rheumatic Valvular Heart Disease–related Mortality, Stratified by Place of Death in Adults in the United States, 1999 to 2023

| **Place of Death** | **Deaths** |
| --- | --- |
| Medical Facility - Inpatient | 49,170 |
| Medical Facility - Outpatient or ER | 6,275 |
| Medical Facility - Dead on Arrival | 396 |
| Medical Facility - Status unknown | 57 |
| Decedent's home | 42,209 |
| Hospice facility | 7,303 |
| Nursing home/long term care | 29,953 |
| Other | 6,334 |
| Place of death unknown | 142 |
| **Total** | 141,839 |
