# Supplementary material for: Mortality involving non-rheumatic valvular heart disease and atrial fibrillation in the United States: Long-term trends and implications
Source: Medicine (Baltimore). 2026 Oct 9;105(41):e51020. doi: 10.1097/MD.0000000000051020 (PMC13657543; doi:10.1097/MD.0000000000051020)
Supplement: Supplementary file 5 [file medi-105-e51020-s005.docx]

**Supplemental Table 4** Overall and Sex‐Stratified Atrial Fibrillation and Non-Rheumatic Valvular Heart Disease–related Age-Adjusted Mortality Rates per 100,000 in Adults in the United States, 1999 to 2023.

| **Year** | **Overall** | **Women** | **Men** |
| --- | --- | --- | --- |
| 1999 | 2.98 (2.87-3.09) | 2.8 (2.67-2.93) | 3.32 (3.12-3.52) |
| 2000 | 3.01 (2.9-3.12) | 2.84 (2.7-2.97) | 3.21 (3.01-3.41) |
| 2001 | 2.95 (2.84-3.06) | 2.83 (2.7-2.97) | 3.14 (2.95-3.33) |
| 2002 | 3.02 (2.91-3.13) | 2.82 (2.69-2.96) | 3.33 (3.13-3.53) |
| 2003 | 3.08 (2.97-3.19) | 2.88 (2.74-3.01) | 3.46 (3.27-3.66) |
| 2004 | 3.16 (3.05-3.27) | 2.9 (2.77-3.04) | 3.61 (3.41-3.81) |
| 2005 | 3.36 (3.25-3.47) | 3.19 (3.05-3.32) | 3.66 (3.46-3.85) |
| 2006 | 3.35 (3.24-3.46) | 3.08 (2.95-3.21) | 3.72 (3.52-3.91) |
| 2007 | 3.6 (3.49-3.72) | 3.28 (3.14-3.41) | 4.07 (3.87-4.27) |
| 2008 | 3.61 (3.5-3.72) | 3.31 (3.17-3.44) | 3.97 (3.77-4.16) |
| 2009 | 3.55 (3.44-3.66) | 3.26 (3.12-3.39) | 3.97 (3.78-4.17) |
| 2010 | 3.97 (3.86-4.09) | 3.56 (3.42-3.7) | 4.63 (4.43-4.84) |
| 2011 | 4.06 (3.94-4.17) | 3.64 (3.5-3.77) | 4.65 (4.45-4.85) |
| 2012 | 4.22 (4.1-4.33) | 3.74 (3.6-3.87) | 4.97 (4.76-5.17) |
| 2013 | 4.62 (4.5-4.74) | 4.16 (4.01-4.3) | 5.31 (5.1-5.52) |
| 2014 | 4.83 (4.71-4.95) | 4.2 (4.05-4.34) | 5.71 (5.5-5.92) |
| 2015 | 4.96 (4.84-5.08) | 4.37 (4.22-4.52) | 5.77 (5.56-5.98) |
| 2016 | 5.26 (5.13-5.38) | 4.59 (4.45-4.74) | 6.25 (6.03-6.47) |
| 2017 | 5.64 (5.52-5.77) | 4.95 (4.79-5.1) | 6.68 (6.46-6.9) |
| 2018 | 5.81 (5.69-5.94) | 5.07 (4.92-5.23) | 6.86 (6.64-7.09) |
| 2019 | 6.03 (5.9-6.16) | 5.13 (4.97-5.28) | 7.24 (7.02-7.47) |
| 2020 | 6.4 (6.27-6.53) | 5.28 (5.13-5.43) | 8.08 (7.85-8.32) |
| 2021 | 7.42 (7.27-7.57) | 6.23 (6.05-6.4) | 9.11 (8.85-9.37) |
| 2022 | 7.29 (7.15-7.43) | 6.1 (5.94-6.26) | 9.01 (8.77-9.26) |
| 2023 | 7.3 (7.16-7.44) | 6.18 (6.01-6.35) | 8.96 (8.71-9.2) |
