# Supplementary material for: Mortality involving non-rheumatic valvular heart disease and atrial fibrillation in the United States: Long-term trends and implications
Source: Medicine (Baltimore). 2026 Oct 9;105(41):e51020. doi: 10.1097/MD.0000000000051020 (PMC13657543; doi:10.1097/MD.0000000000051020)
Supplement: Supplementary file 6 [file medi-105-e51020-s006.docx]

**Supplemental Table 5** Atrial Fibrillation and Non-Rheumatic Valvular Heart Disease–related Age-Adjusted Mortality Rates per 100,000, Stratified by Race in Adults in the United States, 1999 to 2023

| **Year** | **NH White** | **NH Black or African American** | **NH Asian or Pacific Islander** | **NH American Indian or Alaska Native** | **Hispanic or Latino** |
| --- | --- | --- | --- | --- | --- |
| 1999 | 3.23 (3.11-3.36) | 1.16 (0.92-1.44) | 1.65 (1.1-2.36) | Missing | 1.47 (1.1-1.93) |
| 2000 | 3.28 (3.15-3.4) | 1.09 (0.86-1.35) | 1.06 (0.65-1.64) | Missing | 1.32 (0.98-1.72) |
| 2001 | 3.25 (3.13-3.37) | 0.87 (0.67-1.11) | 1.23 (0.78-1.85) | Missing | 1.37 (1.04-1.78) |
| 2002 | 3.3 (3.17-3.42) | 1.19 (0.96-1.47) | 1.23 (0.81-1.81) | Missing | 1.13 (0.83-1.5) |
| 2003 | 3.37 (3.24-3.49) | 1.12 (0.89-1.39) | 1.67 (1.18-2.31) | Missing | 1.39 (1.07-1.79) |
| 2004 | 3.51 (3.38-3.63) | 1.19 (0.96-1.46) | 1.49 (1.03-2.08) | Missing | 0.93 (0.67-1.25) |
| 2005 | 3.72 (3.59-3.85) | 0.93 (0.73-1.17) | 1.39 (0.98-1.93) | Missing | 1.25 (0.96-1.59) |
| 2006 | 3.73 (3.6-3.86) | 1.1 (0.88-1.36) | 1.55 (1.12-2.08) | Missing | 1.01 (0.75-1.32) |
| 2007 | 4.04 (3.9-4.17) | 1.04 (0.83-1.29) | 1.93 (1.45-2.51) | Missing | 1.23 (0.96-1.56) |
| 2008 | 4.03 (3.9-4.16) | 1.16 (0.93-1.39) | 1.18 (0.82-1.64) | Missing | 1.31 (1.03-1.64) |
| 2009 | 3.94 (3.81-4.07) | 1.22 (0.99-1.45) | 1.99 (1.54-2.53) | Missing | 1.65 (1.33-1.97) |
| 2010 | 4.44 (4.31-4.58) | 1.41 (1.16-1.65) | 1.7 (1.29-2.19) | Missing | 1.66 (1.35-1.98) |
| 2011 | 4.57 (4.43-4.7) | 1.4 (1.16-1.65) | 1.83 (1.43-2.32) | Missing | 1.4 (1.13-1.67) |
| 2012 | 4.75 (4.62-4.89) | 1.41 (1.18-1.65) | 1.66 (1.29-2.11) | Missing | 1.97 (1.66-2.29) |
| 2013 | 5.24 (5.1-5.38) | 1.66 (1.41-1.91) | 1.96 (1.57-2.42) | Missing | 1.7 (1.42-1.98) |
| 2014 | 5.45 (5.3-5.59) | 1.71 (1.46-1.96) | 1.91 (1.53-2.34) | Missing | 2.35 (2.03-2.67) |
| 2015 | 5.69 (5.55-5.84) | 1.69 (1.44-1.94) | 1.79 (1.45-2.19) | 3.27 (1.97-5.11) | 2.12 (1.83-2.42) |
| 2016 | 6.05 (5.9-6.19) | 1.74 (1.49-1.98) | 2.32 (1.91-2.72) | Missing | 2.18 (1.89-2.47) |
| 2017 | 6.55 (6.4-6.71) | 1.89 (1.64-2.14) | 2.07 (1.7-2.44) | 3.07 (1.87-4.74) | 2.3 (2.1-2.69) |
| 2018 | 6.7 (6.54-6.85) | 2.17 (1.9-2.44) | 2.58 (2.18-2.99) | Missing | 2.64 (2.33-2.94) |
| 2019 | 6.98 (6.83-7.14) | 2.44 (2.16-2.72) | 2.52 (2.14-2.91) | 3.75 (2.51-5.39) | 2.34 (2.06-2.62) |
| 2020 | 7.45 (7.29-7.61) | 2.65 (2.37-2.94) | 2.2 (1.85-2.54) | 3.57 (2.35-5.19) | 2.84 (2.53-3.14) |
| 2021 | 8.74 (8.56-8.92) | 2.57 (2.28-2.86) | 2.51 (2.14-2.89) | 5.58 (3.96-7.62) | 3.32 (2.99-3.65) |
| 2022 | 8.56 (8.39-8.73) | 2.97 (2.67-3.27) | 2.82 (2.44-3.2) | 4.43 (3.08-6.16) | 2.9 (2.6-3.21) |
| 2023 | 8.65 (8.47-8.82) | 2.88 (2.59-3.17) | 2.91 (2.53-3.29) | 3.31 (2.18-4.81) | 3.07 (2.76-3.37) |

NH = non-Hispanic.
