# Supplementary material for: Mortality involving non-rheumatic valvular heart disease and atrial fibrillation in the United States: Long-term trends and implications
Source: Medicine (Baltimore). 2026 Oct 9;105(41):e51020. doi: 10.1097/MD.0000000000051020 (PMC13657543; doi:10.1097/MD.0000000000051020)
Supplement: Supplementary file 7 [file medi-105-e51020-s007.docx]

**Supplemental Table 6** Atrial Fibrillation and Non-Rheumatic Valvular Heart Disease–related Age-Adjusted Mortality Rates per 100,000, Stratified by Census Region in Adults in the United States, 1999 to 2023.

| **Census Region** | **Year** | **Age Adjusted Rate** |
| --- | --- | --- |
| Northeast | 1999 | 3.47 (3.21-3.73) |
| Northeast | 2000 | 3.44 (3.19-3.69) |
| Northeast | 2001 | 3.36 (3.11-3.61) |
| Northeast | 2002 | 3.45 (3.2-3.7) |
| Northeast | 2003 | 3.54 (3.29-3.79) |
| Northeast | 2004 | 3.65 (3.4-3.9) |
| Northeast | 2005 | 3.41 (3.16-3.65) |
| Northeast | 2006 | 3.6 (3.36-3.85) |
| Northeast | 2007 | 3.82 (3.57-4.07) |
| Northeast | 2008 | 3.88 (3.62-4.13) |
| Northeast | 2009 | 3.94 (3.69-4.2) |
| Northeast | 2010 | 4.19 (3.93-4.45) |
| Northeast | 2011 | 4.25 (3.99-4.51) |
| Northeast | 2012 | 4.24 (3.99-4.5) |
| Northeast | 2013 | 5.03 (4.75-5.31) |
| Northeast | 2014 | 5.14 (4.86-5.43) |
| Northeast | 2015 | 5.27 (4.99-5.55) |
| Northeast | 2016 | 5.38 (5.09-5.66) |
| Northeast | 2017 | 5.87 (5.58-6.16) |
| Northeast | 2018 | 5.74 (5.45-6.02) |
| Northeast | 2019 | 5.95 (5.66-6.24) |
| Northeast | 2020 | 6.65 (6.35-6.95) |
| Northeast | 2021 | 7.78 (7.44-8.12) |
| Northeast | 2022 | 7.63 (7.3-7.96) |
| Northeast | 2023 | 7.75 (7.42-8.08) |
| Midwest | 1999 | 3.04 (2.82-3.27) |
| Midwest | 2000 | 3.07 (2.84-3.29) |
| Midwest | 2001 | 3.02 (2.8-3.24) |
| Midwest | 2002 | 3.05 (2.83-3.27) |
| Midwest | 2003 | 3.18 (2.95-3.4) |
| Midwest | 2004 | 3.07 (2.85-3.29) |
| Midwest | 2005 | 3.4 (3.17-3.63) |
| Midwest | 2006 | 3.35 (3.12-3.57) |
| Midwest | 2007 | 3.68 (3.45-3.92) |
| Midwest | 2008 | 3.78 (3.55-4.02) |
| Midwest | 2009 | 3.48 (3.25-3.7) |
| Midwest | 2010 | 3.88 (3.65-4.12) |
| Midwest | 2011 | 4.27 (4.02-4.51) |
| Midwest | 2012 | 4.31 (4.07-4.55) |
| Midwest | 2013 | 4.89 (4.63-5.15) |
| Midwest | 2014 | 5.09 (4.83-5.35) |
| Midwest | 2015 | 5.28 (5.02-5.54) |
| Midwest | 2016 | 5.85 (5.57-6.13) |
| Midwest | 2017 | 5.95 (5.67-6.23) |
| Midwest | 2018 | 6.34 (6.06-6.63) |
| Midwest | 2019 | 6.91 (6.61-7.2) |
| Midwest | 2020 | 7.2 (6.97-7.57) |
| Midwest | 2021 | 8.12 (7.79-8.45) |
| Midwest | 2022 | 7.7 (7.39-8.01) |
| Midwest | 2023 | 7.89 (7.57-8.2) |
| South | 1999 | 2.18 (2.02-2.34) |
| South | 2000 | 2.22 (2.06-2.38) |
| South | 2001 | 2.26 (2.1-2.42) |
| South | 2002 | 2.31 (2.15-2.47) |
| South | 2003 | 2.33 (2.17-2.49) |
| South | 2004 | 2.49 (2.33-2.66) |
| South | 2005 | 2.65 (2.48-2.81) |
| South | 2006 | 2.53 (2.36-2.69) |
| South | 2007 | 2.69 (2.52-2.86) |
| South | 2008 | 2.61 (2.45-2.77) |
| South | 2009 | 2.73 (2.56-2.89) |
| South | 2010 | 3.1 (2.93-3.28) |
| South | 2011 | 3.01 (2.84-3.17) |
| South | 2012 | 3.23 (3.06-3.4) |
| South | 2013 | 3.41 (3.23-3.58) |
| South | 2014 | 3.71 (3.53-3.89) |
| South | 2015 | 3.63 (3.46-3.8) |
| South | 2016 | 3.82 (3.64-3.99) |
| South | 2017 | 4.36 (4.17-4.54) |
| South | 2018 | 4.54 (4.35-4.72) |
| South | 2019 | 4.64 (4.46-4.83) |
| South | 2020 | 5 (4.81-5.19) |
| South | 2021 | 5.84 (5.63-6.06) |
| South | 2022 | 5.85 (5.65-6.06) |
| South | 2023 | 5.89 (5.68-6.1) |
| West | 1999 | 3.82 (3.54-4.11) |
| West | 2000 | 3.79 (3.51-4.07) |
| West | 2001 | 3.7 (3.43-3.97) |
| West | 2002 | 3.69 (3.42-3.96) |
| West | 2003 | 3.83 (3.56-4.1) |
| West | 2004 | 3.97 (3.69-4.24) |
| West | 2005 | 4.3 (4.02-4.59) |
| West | 2006 | 4.31 (4.04-4.59) |
| West | 2007 | 4.81 (4.52-5.09) |
| West | 2008 | 4.62 (4.34-4.9) |
| West | 2009 | 4.58 (4.3-4.85) |
| West | 2010 | 5.21 (4.93-5.5) |
| West | 2011 | 5.36 (5.07-5.64) |
| West | 2012 | 5.6 (5.37-5.95) |
| West | 2013 | 5.89 (5.6-6.18) |
| West | 2014 | 6.01 (5.72-6.31) |
| West | 2015 | 6.38 (6.09-6.67) |
| West | 2016 | 6.93 (6.62-7.23) |
| West | 2017 | 7.27 (6.96-7.57) |
| West | 2018 | 7.38 (7.07-7.68) |
| West | 2019 | 7.45 (7.15-7.75) |
| West | 2020 | 7.67 (7.36-7.97) |
| West | 2021 | 8.99 (8.65-9.33) |
| West | 2022 | 8.98 (8.65-9.31) |
| West | 2023 | 8.72 (8.39-9.04) |
