# Supplementary material for: Mortality involving non-rheumatic valvular heart disease and atrial fibrillation in the United States: Long-term trends and implications
Source: Medicine (Baltimore). 2026 Oct 9;105(41):e51020. doi: 10.1097/MD.0000000000051020 (PMC13657543; doi:10.1097/MD.0000000000051020)
Supplement: Supplementary file 8 [file medi-105-e51020-s008.docx]

**Supplemental Table 7** Atrial Fibrillation and Non-Rheumatic Valvular Heart Disease–related Age-Adjusted Mortality Rates per 100,000, Stratified by Urban-Rural Classification in Adults in the United States, 1999 to 2020.

| **Year** | **Metropolitan** | **Non-metropolitan** |
| --- | --- | --- |
| 1999 | 3.01 (2.89-3.13) | 2.81 (2.57-3.05) |
| 2000 | 3.06 (2.93-3.18) | 2.89 (2.65-3.14) |
| 2001 | 2.88 (2.76-3) | 3.32 (3.05-3.58) |
| 2002 | 3.07 (2.94-3.19) | 2.76 (2.52-3) |
| 2003 | 3.08 (2.96-3.2) | 3.03 (2.79-3.28) |
| 2004 | 3.1 (2.98-3.22) | 3.52 (3.25-3.79) |
| 2005 | 3.34 (3.22-3.46) | 3.37 (3.11-3.63) |
| 2006 | 3.34 (3.22-3.47) | 3.22 (2.97-3.47) |
| 2007 | 3.48 (3.36-3.61) | 3.95 (3.68-4.23) |
| 2008 | 3.56 (3.44-3.68) | 3.75 (3.48-4.02) |
| 2009 | 3.52 (3.4-3.64) | 3.64 (3.38-3.9) |
| 2010 | 3.94 (3.82-4.07) | 4.08 (3.81-4.36) |
| 2011 | 4.03 (3.91-4.16) | 4.04 (3.77-4.31) |
| 2012 | 4.17 (4.05-4.3) | 4.35 (4.07-4.63) |
| 2013 | 4.58 (4.45-4.71) | 4.73 (4.44-5.02) |
| 2014 | 4.73 (4.59-4.86) | 5.3 (4.99-5.6) |
| 2015 | 4.85 (4.72-4.98) | 5.39 (5.09-5.69) |
| 2016 | 5.2 (5.06-5.33) | 5.5 (5.2-5.81) |
| 2017 | 5.57 (5.44-5.71) | 5.99 (5.67-6.31) |
| 2018 | 5.69 (5.55-5.83) | 6.36 (6.03-6.68) |
| 2019 | 5.87 (5.73-6.01) | 6.68 (6.35-7.01) |
| 2020 | 6.2 (6.06-6.34) | 7.31 (6.97-7.65) |
