# Supplementary material for: Mortality involving non-rheumatic valvular heart disease and atrial fibrillation in the United States: Long-term trends and implications
Source: Medicine (Baltimore). 2026 Oct 9;105(41):e51020. doi: 10.1097/MD.0000000000051020 (PMC13657543; doi:10.1097/MD.0000000000051020)
Supplement: Supplementary file 9 [file medi-105-e51020-s009.docx]

**Supplemental Table 8** Atrial Fibrillation and Non-Rheumatic Valvular Heart Disease–related Crude Mortality Rates per 100,000, Stratified by Age in Adults in the United States, 1999 to 2023.

| **Year** | **Age 45-64 Years** | **Age 65+ Years** |
| --- | --- | --- |
| 1999 | 0.13 (0.1-0.16) | 7.89 (7.59-8.18) |
| 2000 | 0.12 (0.1-0.15) | 7.99 (7.69-8.29) |
| 2001 | 0.11 (0.08-0.14) | 8.01 (7.71-8.3) |
| 2002 | 0.12 (0.1-0.15) | 8.15 (7.85-8.45) |
| 2003 | 0.1 (0.08-0.13) | 8.44 (8.14-8.74) |
| 2004 | 0.11 (0.09-0.14) | 8.73 (8.43-9.04) |
| 2005 | 0.15 (0.12-0.18) | 9.16 (8.85-9.47) |
| 2006 | 0.14 (0.12-0.17) | 9.23 (8.92-9.54) |
| 2007 | 0.13 (0.11-0.16) | 10.08 (9.76-10.4) |
| 2008 | 0.15 (0.12-0.18) | 10 (9.68-10.31) |
| 2009 | 0.15 (0.12-0.17) | 9.95 (9.64-10.26) |
| 2010 | 0.19 (0.16-0.21) | 11 (10.68-11.33) |
| 2011 | 0.14 (0.12-0.17) | 11.48 (11.15-11.81) |
| 2012 | 0.19 (0.16-0.22) | 11.7 (11.38-12.03) |
| 2013 | 0.1 (0.16-0.22) | 12.62 (12.29-12.95) |
| 2014 | 0.21 (0.18-0.24) | 12.99 (12.67-13.32) |
| 2015 | 0.23 (0.19-0.26) | 13.23 (12.9-13.55) |
| 2016 | 0.27 (0.23-0.3) | 13.91 (13.58-14.24) |
| 2017 | 0.32 (0.28-0.36) | 14.71 (14.38-15.04) |
| 2018 | 0.28 (0.25-0.32) | 15.05 (14.72-15.38) |
| 2019 | 0.34 (0.3-0.38) | 15.37 (15.04-15.7) |
| 2020 | 0.4 (0.36-0.45) | 16.11 (15.78-16.45) |
| 2021 | 0.4 (0.36-0.45) | 17.48 (17.13-17.83) |
| 2022 | 0.46 (0.41-0.51) | 17.76 (17.41-18.1) |
| 2023 | 0.43 (0.39-0.48) | 17.22 (16.88-17.55) |
