# Supplementary material for: Mortality involving non-rheumatic valvular heart disease and atrial fibrillation in the United States: Long-term trends and implications
Source: Medicine (Baltimore). 2026 Oct 9;105(41):e51020. doi: 10.1097/MD.0000000000051020 (PMC13657543; doi:10.1097/MD.0000000000051020)
Supplement: Supplementary file 10 [file medi-105-e51020-s010.docx]

**Supplemental Table 10** Atrial Fibrillation and Non-Rheumatic Valvular Heart Disease –related Age-Adjusted Mortality Rates per 100,000, Stratified by States in Adults in the United States, 2021 to 2023.

| **State** | **Age Adjusted Rate** |
| --- | --- |
| Alabama | 3.4 (2.96-3.87) |
| Alaska | 6.54 (4.58-9.06) |
| Arizona | 5.99 (5.52-6.47) |
| Arkansas | 4.25 (3.6-4.9) |
| California | 6.93 (6.69-7.17) |
| Colorado | 11.4 (10.54-12.25) |
| Connecticut | 5.32 (4.69-5.95) |
| Delaware | 9.83 (8.18-11.48) |
| District of Columbia | 4.34 (2.95-6.16) |
| Florida | 5.11 (4.88-5.34) |
| Georgia | 2.62 (2.32-2.93) |
| Hawaii | 5.24 (4.3-6.18) |
| Idaho | 12.48 (10.97-13.99) |
| Illinois | 4.18 (3.86-4.49) |
| Indiana | 6.44 (5.89-6.99) |
| Iowa | 10.85 (9.89-11.81) |
| Kansas | 6.93 (6.09-7.77) |
| Kentucky | 6.93 (6.23-7.62) |
| Louisiana | 4.02 (3.48-4.56) |
| Maine | 11.04 (9.64-12.43) |
| Maryland | 10.23 (9.51-10.95) |
| Massachusetts | 7.14 (6.6-7.68) |
| Michigan | 5.49 (5.09-5.89) |
| Minnesota | 17.75 (16.79-18.7) |
| Mississippi | 5.24 (4.49-5.99) |
| Missouri | 6.46 (5.9-7.01) |
| Montana | 8.68 (7.18-10.19) |
| Nebraska | 13.44 (12.01-14.87) |
| Nevada | 6.11 (5.29-6.94) |
| New Hampshire | 10 (8.6-11.4) |
| New Jersey | 7.18 (6.7-7.65) |
| New Mexico | 2.63 (2.07-3.29) |
| New York | 6.02 (5.73-6.31) |
| North Carolina | 6.35 (5.91-6.79) |
| North Dakota | 7.1 (5.56-8.93) |
| Ohio | 6.54 (6.14-6.94) |
| Oklahoma | 7.01 (6.25-7.77) |
| Oregon | 21.71 (20.47-22.95) |
| Pennsylvania | 10.05 (9.6-10.5) |
| Rhode Island | 9.56 (8.05-11.07) |
| South Carolina | 7.79 (7.11-8.47) |
| South Dakota | 12.35 (10.36-14.33) |
| Tennessee | 7.12 (6.54-7.69) |
| Texas | 5.93 (5.64-6.22) |
| Utah | 4.44 (3.63-5.24) |
| Vermont | 20.7 (17.79-23.61) |
| Virginia | 7.46 (6.93-7.98) |
| Washington | 17.31 (16.44-18.18) |
| West Virginia | 6.02 (5.07-6.97) |
| Wisconsin | 12.47 (11.69-13.25) |
| Wyoming | 12.82 (10.32-15.74) |
